# Host and parasite responses in human diffuse cutaneous leishmaniasis caused by *L*. *amazonensis*

**DOI:** 10.1371/journal.pntd.0007152

**Published:** 2019-03-07

**Authors:** Stephen M. Christensen, Ashton T. Belew, Najib M. El-Sayed, Wagner L. Tafuri, Fernando T. Silveira, David M. Mosser

**Affiliations:** 1 Department of Cell Biology and Molecular Genetics and the Maryland Pathogen Research Institute, University of Maryland, College Park, MD United States of America; 2 Center for Bioinformatics and Computational Biology, University of Maryland, College Park, MD United States of America; 3 Departamento de Patologia Geral, Universidade Federal de Minas Geras, Belo Horizonte, Brazil; 4 Evandro Chagas Institute, Tropical Medicine Nucleus, Federal University of Pará, Belém, PA Brazil; Institut Pasteur de Tunis, TUNISIA

## Abstract

Diffuse cutaneous leishmaniasis (DCL) is a rare form of leishmaniasis where parasites grow uncontrolled in diffuse lesions across the skin. Meta-transcriptomic analysis of biopsies from DCL patients infected with *Leishmania amazonensis* demonstrated an infiltration of atypical B cells producing a surprising preponderance of the IgG4 isotype. DCL lesions contained minimal CD8^+^ T cell transcripts and no evidence of persistent T_H_2 responses. Whereas localized disease exhibited activated (so-called M1) macrophage presence, transcripts in DCL suggested a regulatory macrophage (R-Mϕ) phenotype with higher levels of ABCB5, DCSTAMP, SPP1, SLAMF9, PPARG, MMPs, and TM4SF19. The high levels of parasite transcripts in DCL and the remarkable uniformity among patients afforded a unique opportunity to study parasite gene expression in this disease. Patterns of parasite gene expression in DCL more closely resembled *in vitro* parasite growth in resting macrophages, in the absence of T cells. In contrast, parasite gene expression in LCL revealed 336 parasite genes that were differently upregulated, relative to DCL and in vitro macrophage growth, and these transcripts may represent transcripts that are produced by the parasite in response to host immune pressure.

## Author summary

The rare diffuse form of cutaneous leishmaniasis (DCL) manifests as non-ulcerative lesions across the skin. This disease is caused by the parasite *Leishmania amazonensis* that grows uncontrollably in lesions. A complete picture of host-pathogen interactions is not fully understood in DCL. We used RNA-sequencing of patient biopsies to observe host and parasite transcriptomes within this disease. In established chronic disease we discovered (1) atypical B cells producing a surprisingly dominant IgG4 isotype infiltrated lesions, (2) an absence of cytotoxic and T_H_2 T cell responses, and (3) host macrophage responses representing a regulatory macrophage phenotype that struggles to eliminate intracellular pathogens such as *Leishmania*. The cellular phenotypes and activation states identified in this work could be targeted for better therapeutics and vaccines for leishmaniasis. High parasite numbers in lesions allowed us to identify highly expressed parasite genes in diffuse and localized disease, again serving as potential therapeutic or vaccine targets.

## Introduction

Parasites in the genus *Leishmania* spp cause the spectral disease leishmaniasis, which can range from self-healing cutaneous lesions to a fatal, visceral form of disease [1,2]. Manifestations of cutaneous leishmaniasis can depend on both the parasite species and host immune responses. American tegumentary leishmaniasis (ATL) affects 0.7–1.2 million people per year and is endemic in 18 countries [3]. In Brazil, *Leishmania (Viannia) braziliensis* and *Leishmania (Leishmania) amazonensis*, are considered the most epidemiologically relevant species, due to their wide geographic distribution. ATL can present in many different clinical forms, but they are classically described in four basic categories: localized cutaneous leishmaniasis (LCL); mucocutaneous leishmaniasis (MCL), disseminated leishmaniasis (DL) and anergic diffuse cutaneous leishmaniasis (DCL) [4]. LCL caused by *Leishmania braziliensis* infections typically result in a single dermal lesion, with small numbers of parasites and a strong delayed-type hypersensitivity (DTH) response [5,6]. Roughly 3–5% of these infections can progress to the disfiguring mucocutaneous form of the disease [7]. *L*. *amazonensis* also causes cutaneous disease, but in contrast to *L*. *braziliensis*, it can sometimes manifest as diffuse cutaneous leishmaniasis (DCL) [8,9]. In this rare form of the disease, parasites grow uncontrolled in lesions diffuse across the skin. Patients with DCL typically lack a DTH response [4] and are refractory to chemotherapy [10]. While the morphology and pathology of diffuse cutaneous lesions has been studied [4], the underlying causes are not well understood.

The majority of leishmaniasis research has focused on phagocytic cell killing of parasites and the influence that T cells and their products have on this process. This focus is certainly warranted, yet research has increasingly shown the importance of other cells in the control or persistence of disease. Infiltration of B cells in lesions has previously been demonstrated [11–13], but studies on B cell subsets and their contribution to parasite persistence or killing have pointed to complex and varied roles for immunoglobulin in *Leishmania* infection. Vaccination of dogs [14] and primates [15] with recombinant A2 antigen from *L*. *infantum* induced IgG2a antibodies whose levels correlated with reduced parasite burdens, implying a protective role for parasite-specific IgG. Consistent with this, IgG has been shown to be protective against a variety of intracellular pathogens [16]. However, the interaction of IgG-opsonized *Leishmania* parasites with macrophage Fcγ receptors induces IL-10 production [17,18] and prevents parasite eradication in mice [11,12,19,20]. In humans, levels of serum IgG vary depending on parasite species and clinical manifestation. High levels of serum IgG have previously been associated with *L*. *amazonensis* infections in DCL patients and also in *L*. *chagasi* infections in visceral leishmaniasis [4,12,21,22].

Macrophages act as the primary host cells in which parasites reside and replicate. During experimental murine infections, infected macrophages undergo transcriptional and morphological changes that allow for parasite survival, including inhibited iNOS, TNF-α, and IL-12 in concert with increases in IL-10, PGE2, and TGF-β expression [23]. Immune signals from T cells can mitigate parasite manipulations and research in mice has demonstrated a clear role for T_H_1 responses and IFN-γ, TNF-α, and iNOS in parasite clearance. Conversely, T_H_2 responses (IL-4, IL-13) are associated with parasite persistence and disease progression in mice [24,25]. A similarly clear dichotomy has not been confirmed in humans. Whereas inflammatory T_H_1 effectors and subsequent macrophage activation have been associated with a restriction of parasite replication in humans [2], the search for T_H_2 cytokines and specifically downstream alternative macrophage activation markers has been less successful [26]. Our previous studies [27] and that of our colleagues [28] showed a significant T_H_1 response in localized ATL caused by *L*. *braziliansis*. L. amazonensis infections, in contrast, have been associated with T cell hyporesponsiveness [4,8]. In this work, we aimed to assess host and parasite responses in the diffuse form of cutaneous leishmaniasis. We contrast previous LCL results [27] with newly collected data from DCL patients infected with *L*. *amazonensis* and provide an in-depth view of host and parasite responses in this rare and unusual manifestation of leishmaniasis.

## Materials and methods

### Ethics statement

This study was approved by the Ethics Committee in Human Research of the Evandro Chagas Institute (Surveillance Secretary of Health, Ministry of Health, Brazil) and Brazil Platform, under protocol number 102.885/2012. All patients enrolled in this study were informed about the study and signed a free-consent form in accordance with the principles of the Declaration of Helsinki. This study was approved by the Ethics Committees of the University of Maryland (College Park)(925281–2).

### Patients and procedures

All diffuse cutaneous leishmaniasis (DCL) patients were seen in the ambulatory care suite at the Evandro Chagas Institute, Professor Ralph Lainson Laboratory of Leishmaniasis, Ananindeua, Pará Brazil. All of the patients had a confirmed diagnosis of DCL and all had received prior treatments for DCL. Biopsies were collected at the border of the lesions using a 4 mm punch. Patients consisted of 5 males and 1 female with illness duration ranging from 14 to 35 years and age ranging from 15–50 (S1 Table). Healthy (uninfected) and localized cutaneous (LCL) skin samples were taken as previously described [27,28].

### RNA isolation and cDNA library preparation

Samples were placed in RNA later and homogenized using a rotor-stator. Total RNA was isolated using the Trizol extraction and the RNeasy Plus Kit from Qiagen. RNA integrity was assessed using an Agilent 2100 bioanalyzer. Poly(A)^+^-enriched cDNA libraries were generated using the Illumina TruSeq Sample Preparation kit (San Diego, CA) and checked for quality and quantity using the bioanalyzer and qPCR (KAPA Biosystems).

### RNA-seq data generation, pre-processing, and quality trimming

Paired end reads (100 bp) were obtained using the Illumina HiSeq 1500 platform. Trimmomatic [29] was used to remove any remaining Illumina adapter sequences from reads and to trim bases off the start or the end of a read when the quality score fell below a threshold of 20. Sequence quality metrics were assessed using FastQC [http://www.bioinformatics.babraham.ac.uk/projects/fastqc/].

### Mapping cDNA fragments to the reference genome, abundance estimation, and data normalization

TopHat (v 2.0.13) [30] was used to align reads to the applicable genome(s) with each genome alignment performed independently. Reads from healthy, early infection, and late infection skin samples were aligned to the human genome (v. hg19/GRCh37) obtained from the UCSC genome browser (http://genome.ucsc.edu) or the respective parasite genomes (LCL *L*. *braziliensis* v. MHOM/BR/75M2904; DCL: *L*. *mexicana* v. MHOM/GT/2001/U1103) obtained from the TriTrypDB database (www.tritrypdb.org). Parasite reads from DCL patients were mapped to the *L*. *mexicana* genome, the most closely related well-annotated genome available. Two mismatches per read were permitted (default TopHat parameter) and reads were allowed to map only to a single locus (TopHat option–g 1). Additionally, gene model annotations were provided for the mapping (TopHat option–G) with limitations on the identification of novel splice junctions (TopHat option–no-novel-juncs). The abundance of reads mapping to each gene feature in the aligned genome was determined using HTSeq [31]. The resulting count table was restricted to protein-coding genes (20,956 genes for human, 8,556 genes for *L*. *braziliensis*, and 8,246 genes for *L*. *mexicana*). Non-expressed and weakly expressed genes, defined as having less than 1 read per million in n of the samples, where n is the size of the smallest group of replicates [32] (here n = 6), were removed prior to subsequent analyses, resulting in count tables of 15,528 genes (human), 8,556 genes (*L*. *braziliensis*), and 8,246 genes (*L*. *amazonensis/mexicana*).

### Immunoglobulin mapping and analysis

Using filtered and trimmed sequences from the aforementioned pre-processing, reads were aligned using miXCR and pRESTO [33,34]. After preparation, sequences were collapsed and submitted to the ImMunoGeneTics database (IMGT) HighV-QUEST web server for gene annotation and analysis [35,36]. IMGT output was analyzed using in house scripts and bcREP [37].

### Global data assessment, visualization and differential expression analysis

Quantile normalization was applied to all human samples [38] and data were log2-transformed. Multiple approaches were used to evaluate replicates and to visualize the relationships between samples, including Pearson correlation and Principal Component Analysis (PCA). Limma (a Bioconductor package) was used to conduct differential expression analyses [39]. The voom module was used to transform the data based on observational level weights derived from the mean-variance relationship prior to statistical modeling [40]. Pairwise contrasts were done within limma to identify differentially expressed (DE) genes between conditions. Genes with a Benjamini-Hochberg (BH) multiple-testing adjusted *P* value of < 0.05 were defined as differentially expressed. For visualization of absolute gene expression, human and parasite genes were normalized by reads per kilobase per million reads (RPKM) using in house scripts and the given gene lengths from UCSC genome browser or TriTrypDB. All figures noted RPKM used these normalized values. Components of our statistical pipeline, named cbcbSEQ, can be accessed on GitHub (https://github.com/kokrah/cbcbSEQ/).

## Results

### Immunoglobulin transcripts in DCL lesions

RNA-seq was performed on biopsies from six DCL patients infected with *Leishmania amazonensis*. The age of the five male and 1 female patients ranged from 15 to 50, and the duration of illness ranged from 14 to 35 years (S1 Table). A principal component analysis of the host transcriptional response to the infection revealed similarity among all 6 patients and a marked separation from healthy controls and LCL infections (S1A Fig). Pearson correlations of RPKMs confirmed the similarity (0.42–0.95) among host responses of the six DCL patients (S1B Fig) despite substantial differences in age and illness duration. Differential expression comparisons with healthy patients (fold change ≥ 2, adjusted p-value < 0.05) revealed 2420 upregulated host genes (S1C Fig) and 2846 downregulated genes (S1D Fig) in DCL patients. A comparison with previously sequenced LCL lesions demonstrated that DCL and LCL share 1485 upregulated and 1475 downregulated genes (S1C and S1D Fig).

**Fig 1:**
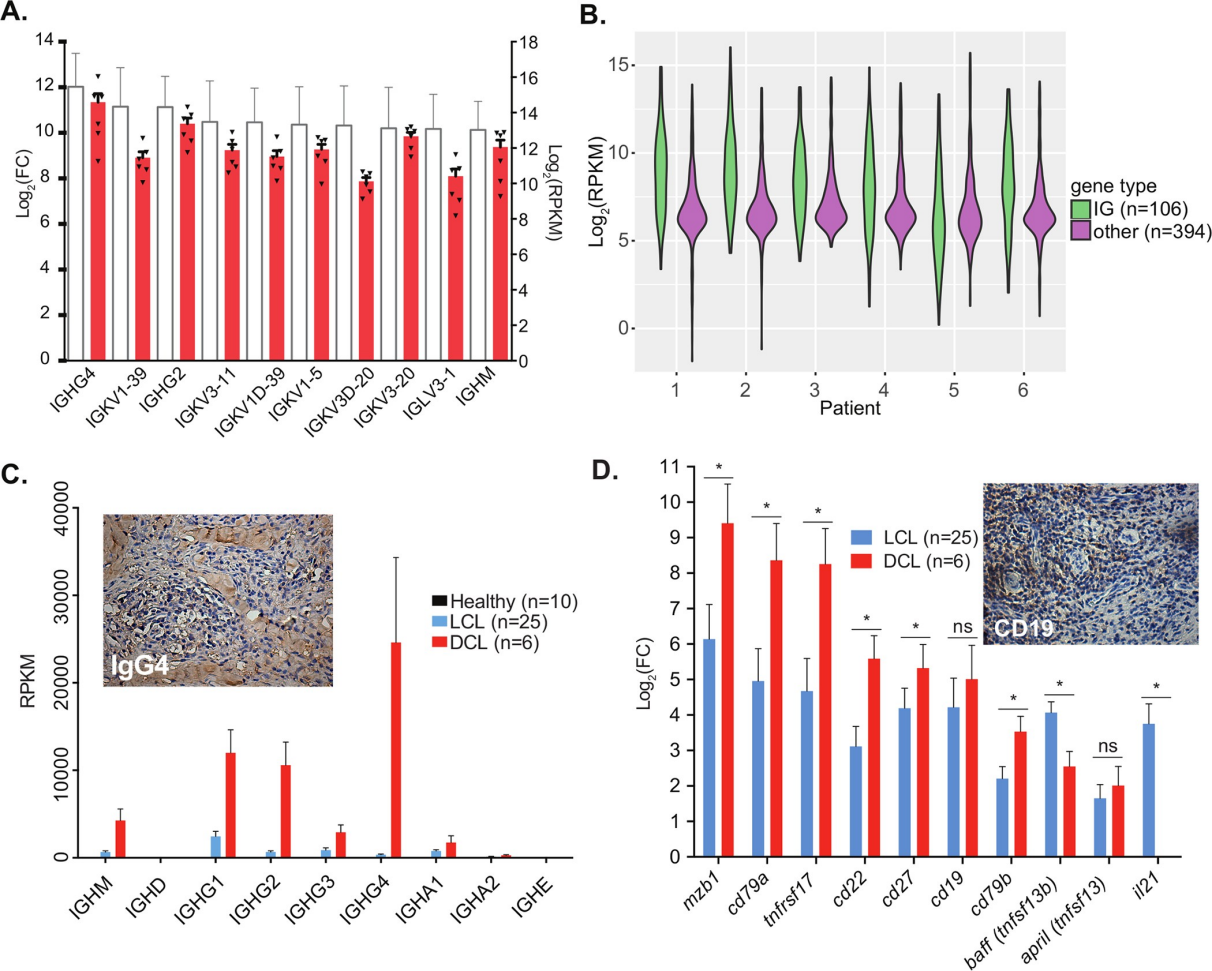
Immunoglobulin transcripts are significantly upregulated in DCL lesions. **(A)** Bars show the fold-change (left y-axis) and RPKM (right y-axis) for the top ten upregulated genes (mean plus SEM), with each individual patient sample RPKM represented by a black triangle. **(B)** A violin plot shows the log_2_(RPKM) for the top 500 genes expressed by the host for each DCL patient. The 500 genes are separated into 106 mmunoglobulin (IG) genes (teal) and 394 genes labeled ‘other’ (purple) **(C)** Bars represent the expression (RPKM mean plus SEM) for each immunoglobulin isotype in healthy skin (black), LCL lesions (blue), and DCL lesions (red). The inset shows immunohistochemical staining of IgG4 in DCL lesions. **(D)** Bars represent log2 fold-changes of B cell markers in LCL (blue) and DCL (red) compared to healthy skin (fold change ≥ 2, adjusted p-value < 0.05). Significant differences between LCL and DCL are marked (*, fold change ≥ 2, adjusted p-value < 0.05). The inset shows immunohistochemical staining of the B cell marker CD19 in DCL lesions.

An examination of host transcripts in DCL lesions pointed to two unusual aspects of the host immune response to this intracellular parasite. The first was the high level of transcripts encoding immunoglobulin fragments and the second was the paucity of transcripts that would likely be expressed by cytotoxic T cells. The top 10 most highly upregulated host transcripts in all six patient biopsies relative to healthy skin encoded immunoglobulin fragments (Fig 1A), and a high degree of consensus existed among the six individual patient RPKMs (Fig 1A, individual triangles within red bars). In fact 90 of the top 100 most highly upregulated transcripts in DCL lesions were immunoglobulin transcripts, and four of the other top 100 transcripts were related to B cells (S2 Table). The expression levels of immunoglobulin genes were high in all six patients and made up >20% of the top 500 expressed host genes (Fig 1B). Due to the mass upregulation of immunoglobulin transcripts, we subsequently analyzed immunoglobulin isotype gene usage and noted significant differences in Ig transcript levels in DCL patient lesions relative to healthy controls and localized (LCL) lesions (Fig 1C). DCL patient lesions expressed high levels of all IgG isotypes (IgG1, IgG2, IgG3 and IgG4), with IgG4 unexpectedly accounting for an average of 40% of the immunoglobulin repertoire (Fig 1C). LCL patients, in contrast, exhibited lower levels of all immunoglobulin isotype transcripts, and IgG1 was the most highly expressed isotype (Fig 1C). IgG4 levels were not different from uninfected controls. Subsequent staining for IgG4 antibody revealed high levels of this immunoglobulin isotype in DCL lesions (Fig 1C, inset).

In addition to upregulated immunoglobulin transcripts, a marked increase in B cell-related transcripts was observed in DCL lesions compared to healthy skin. Transcripts for 9 major B cell-related markers were upregulated in DCL patients, including MZB1, CD79A, TNFRSF17, CD22, CD27, CD19, CD79b, BAFF, and APRIL (Fig 1D). Six of these nine were also significantly upregulated in DCL relative to LCL. Histology from diffuse lesions confirms the infiltration of B cells, with positive CD19 (Fig 1D, inset) staining in DCL lesions.

Using MiXCR and the bioconductor package bcRep [33,37], we observed an enrichment of specific V-J combinations and V gene usage, suggestive of an oligoclonal response in DCL patient lesions. A chord diagram of average heavy chain V-J combination frequency in DCL patients demonstrated a specific immunoglobulin gene selection response limited to 25% of IGHV genes used at a frequency greater than 1% (S2 Fig). Among heavy chain V genes, just 25 genes were used at a frequency greater than 1% (S3A Fig). The heavy chain V genes most represented in DCL included IGHV1-69, IGHV3-30, IGHV3-23, and IGHV4-34. In the same manner, kappa light chain V gene usage consisted mainly of 22 genes, with IGKV1-5 as the most used kappa V gene (>12%) (S3B Fig). Lastly, lambda light chain V gene usage was limited to 18 genes, all of which made up more than 92% of lambda light chain V gene transcripts, with IGLV2-14 as the most used lambda V gene (S3C Fig).

### Altered cytotoxic T cell responses in DCL lesions

**Fig 2:**
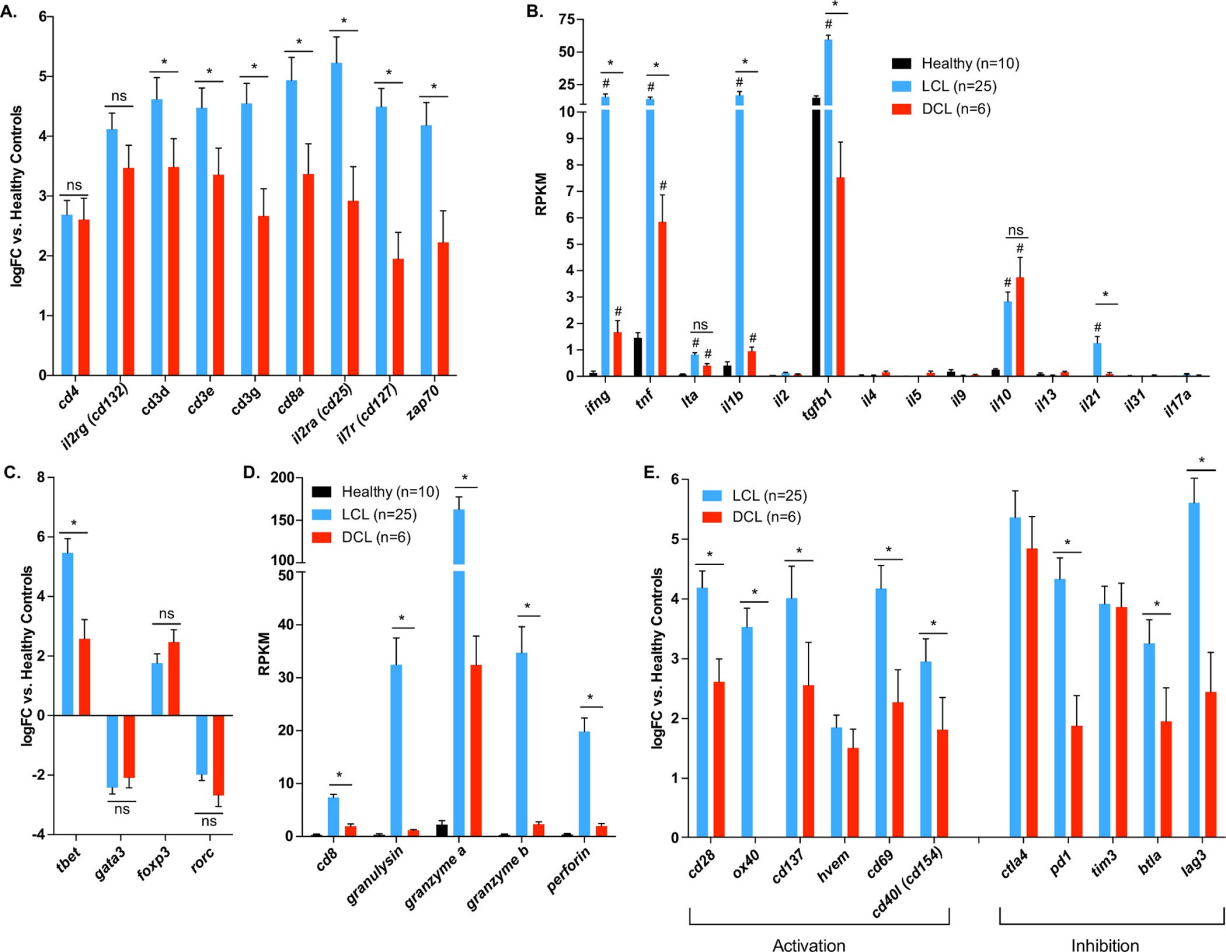
Altered cytotoxic T cell responses in DCL lesions. **(A)** Bars represent the log_2_ fold-change of T cell markers in LCL (blue) and DCL (red) patients (mean plus sem, p < 0.05) relative to healthy patients. Differences between LCL and DCL are indicated (*adjusted p-value < 0.05, ns = not significant). **(B)** Bars show average RPKM values (plus SEM) for healthy (black), LCL (blue), and DCL (red) for cytokines involved in T_H_1 (*ifng*, *tnf*, *lta*, *il1b*, *il2*), T_H_2 (*tgfb1*, *il4*, *il5*, *il9*, *il10*, *il13*, *il21*, *il31*), and T_H_17 (*il17a*) responses. Significant differences versus healthy (#,adjusted p-value < 0.05) and between LCL and DCL (*adjusted p-value < 0.05) are indicated. **(C)** Bars represent the log_2_ fold-change of T cell transcription factors in LCL (blue) and DCL (red) patients (mean plus sem, p < 0.05) relative to healthy patients. Differences between LCL and DCL are indicated (*adjusted p-value < 0.05, ns = not significant). **(D)** Bars show mean RPKM values (plus SEM) for healthy (black), LCL (blue), and DCL (red) for CD8 and cytotoxic T lymphocyte effector molecules. Significant differences between LCL and DCL are indicated (*adjusted p-value < 0.05). **(E)** Bars represent the log_2_ fold-change of T cell activation and inhibition markers in LCL (blue) and DCL (red) patients (mean plus sem, p < 0.05) relative to healthy patients. Differences between LCL and DCL are indicated (*adjusted p-value < 0.05, ns = not significant).

T cell responses in DCL lesions were examined and compared to healthy controls and lesions from 25 previously analyzed LCL patients [27]. T cell markers were selected based on the literature [41]. DCL and LCL patients expressed similar levels of transcripts for CD4 and CD132 (IL2Rg), but DCL lesions contained lower transcript levels for all three CD3 chains, CD127 (IL-7r), and zap70 (Fig 2A). In DCL lesions, there was a significant reduction in the T_H_1-associated transcripts for IFNγ, TNF, and IL-1β relative to LCL (Fig 2B). Expression of the canonical T_H_2-associated transcripts, IL-4, IL5, and IL-13 were all low and not different from uninfected controls (Fig 2B). Surprisingly, IL-10 transcript levels were comparable in both diseases (Fig 2B). DCL also expressed significantly reduced upregulation of the T_H_1 transcription factor tbet versus LCL, but similar levels for gata3, foxp3, and rorc (Fig 2C).

There was a significant reduction in CD8A transcripts in DCL lesions relative to LCL (Fig 2D). Furthermore, transcripts for the cytotoxic effector molecules granulysin, granzyme A, granzyme B, and perforin were all significantly diminished in DCL lesions compared to LCL (Fig 2D).

Various markers of T cell activation, including transcripts for CD28, OX40, CD137, CD69, and CD40L, were decreased in DCL relative to LCL (Fig 2E). However, the expression of inhibitory signaling molecules indicative of anergy, including PD-1, BTLA, and LAG3, was also significantly lower in DCL (Fig 2E).

### Altered macrophage responses in DCL lesions exhibit regulatory characteristics

**Fig 3:**
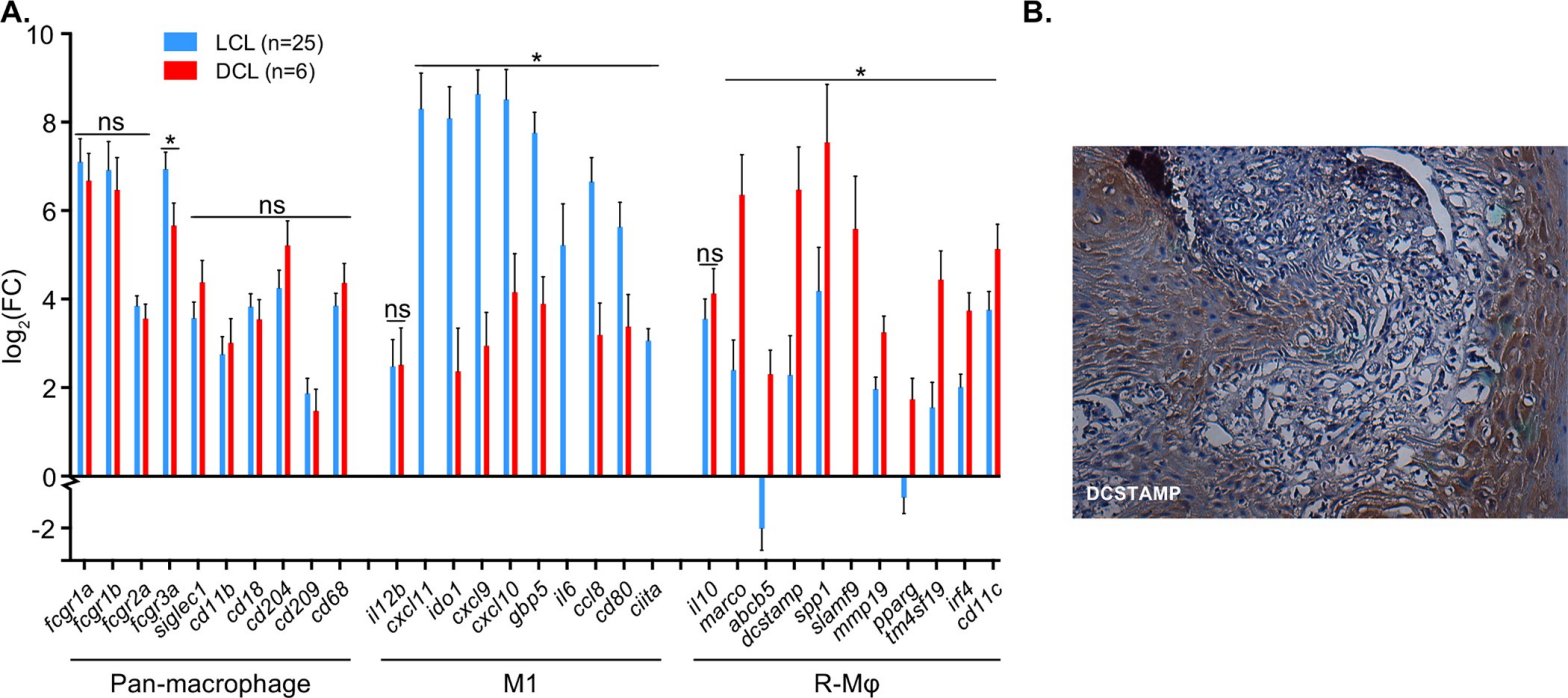
Altered macrophage responses in DCL lesions exhibit regulatory characteristics. **(A)** Pan-macrophage, M1 macrophage, and regulatory macrophage (R-MΦ) transcripts in LCL (blue) and DCL (red) expressed as fold change (log_2_(mean) plus sem) relative to healthy controls. Statistical differences between LCL and DCL are designated (*, adjusted p-value < 0.05; ns = not significant) **(B)** Immunohistochemical staining of DCSTAMP in DCL lesions.

Macrophage transcripts in DCL were quantified and compared to transcripts in LCL. Both DCL and LCL lesions had a significant upregulation of pan-macrophage markers relative to healthy skin (selected based on the literature) [42–44]. The expression of genes typically expressed on macrophages, including FCGR1A, FCGR1B, CD11b, CD18, CD204, and CD68, was comparable in LCL and DCL (Fig 3A, designated ns). Markers of macrophage activation states, however, were significantly different between DCL and LCL.

Data from other work in our lab found that human macrophages stimulated *in vitro* with LPS (so-called M1), expressed a different transcriptome from macrophages stimulated with LPS plus immune complexes (LPS+IC). The former exhibited an inflammatory phenotype whereas the later assumed a regulatory phenotype (R-MΦ) (manuscript in preparation). These R-Mϕs downregulated inflammatory genes and upregulated anti-inflammatory and growth-related genes. We hypothesized that the inflammatory microenvironment in LCL would partially mirror expression of M1 macrophages, while the increased presence of immunoglobulin in the DCL microenvironment would partially mirror R-MΦ expression. R-MΦs generated *in vitro* (LPS+IC) significantly downregulated 271 LPS-induced genes. Of those 271, more than half (146) were significantly downregulated in DCL relative to LCL (S3 Table), including CXCL9, CXCL10, CXCL11, IDO1, GBP5, IL6, CCL8, CD80, and CIITA (Fig 3A). One of the surprising exceptions to this was IL-12β, which is similarly expressed in LCL and DCL lesions (Fig 3A, designated ns). Conversely, R-MΦs significantly upregulated 925 genes relative to LPS-stimulation, 90 of which showed significantly higher expression in DCL compared to LCL (S4 Table). DCL macrophages and R-MΦs similarly upregulated genes, including ABCB5, DCSTAMP, SPP1, SLAMF9, MMP19, PPARG, TM4SF19, IRF4, and CD11c (Fig 3A). A marker of marginal zone (MZ) macrophages (MARCO) was also expressed significantly higher in DCL compared to LCL (Fig 3A). Histology confirmed the presence of DCSTAMP in DCL lesions (Fig 3B). Transcripts encoding the anti-inflammatory cytokine IL-10 were unexpectedly equally upregulated in LCL and DCL (Fig 3A).

Along with the lack of transcripts for IL-4, IL-5, IL-13 in DCL lesions (Fig 2B), biomarkers and chemokines that have been associated with human M2a macrophages were also low in DCL. Transcripts encoding CCR3, CCR4, CCR8, CXCR4, IFNGR1, IFNGR2, IL4RA, IL17BR, IL1RL1, and TSLPR were not different between DCL and LCL (S4 Fig).

### *L*. *amazonensis* gene expression in DCL lesions

**Fig 4:**
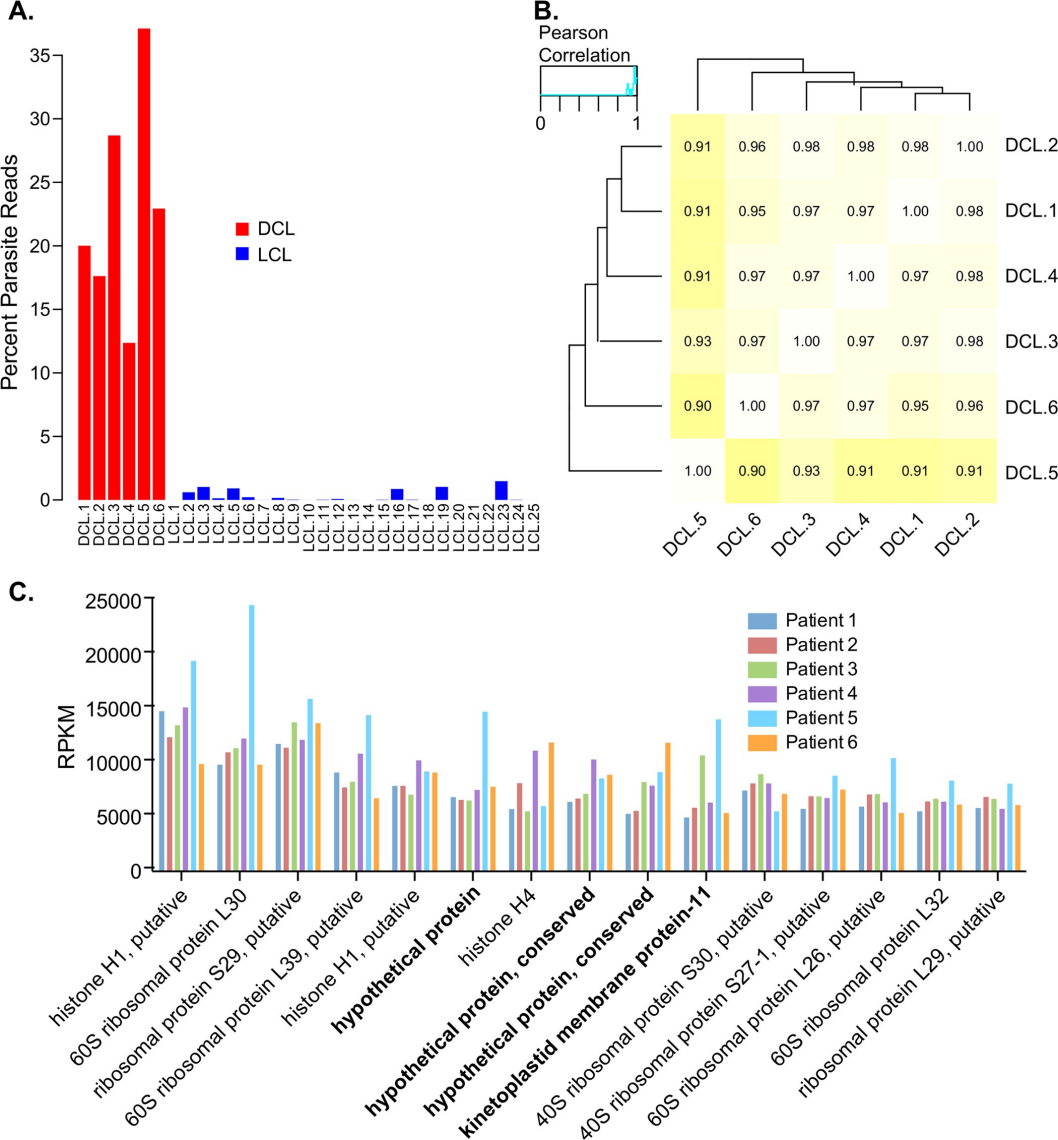
*L*. *amazonensis* gene expression in DCL lesions. **(A)** Bars represent the percent of reads that mapped to parasite genomes in DCL (red) and LCL (blue). **(B)** A heatmap indicating Pearson correlations between the 6 DCL patients. **(C)** Bars represent RPKMs for the top 15 genes expressed by *L*. *amazonensis* in 6 DCL patients. Each patient is designated by a different color.

A high percentage (10–30%) of the total reads in DCL lesions mapped to the parasite genome (Fig 4A, red bars). In contrast, the percentage of reads that mapped to the *L*. *braziliensis* genome in LCL was below 2% (Fig 4A, blue bars). Parasite steady-state transcript levels in diffuse lesions displayed a high degree of patient-to-patient uniformity with Pearson correlations of *L*. *amazonensis* RPKMs greater than 0.9 (Fig 4B). Fig 4C shows the 15 most highly expressed parasite genes and the uniformity of expression in each of the six patients. These top 15 transcripts consisted mainly of ribosomal and histone proteins, but also contained transcripts for hypothetical proteins and the known virulence factor kinetoplastid membrane protein-11. A deeper look at the top 500 expressed parasite genes (S5 Table) revealed 183 hypothetical proteins (>35%) and multiple predicted or known peptidases and heat shock proteins.

**Fig 5:**
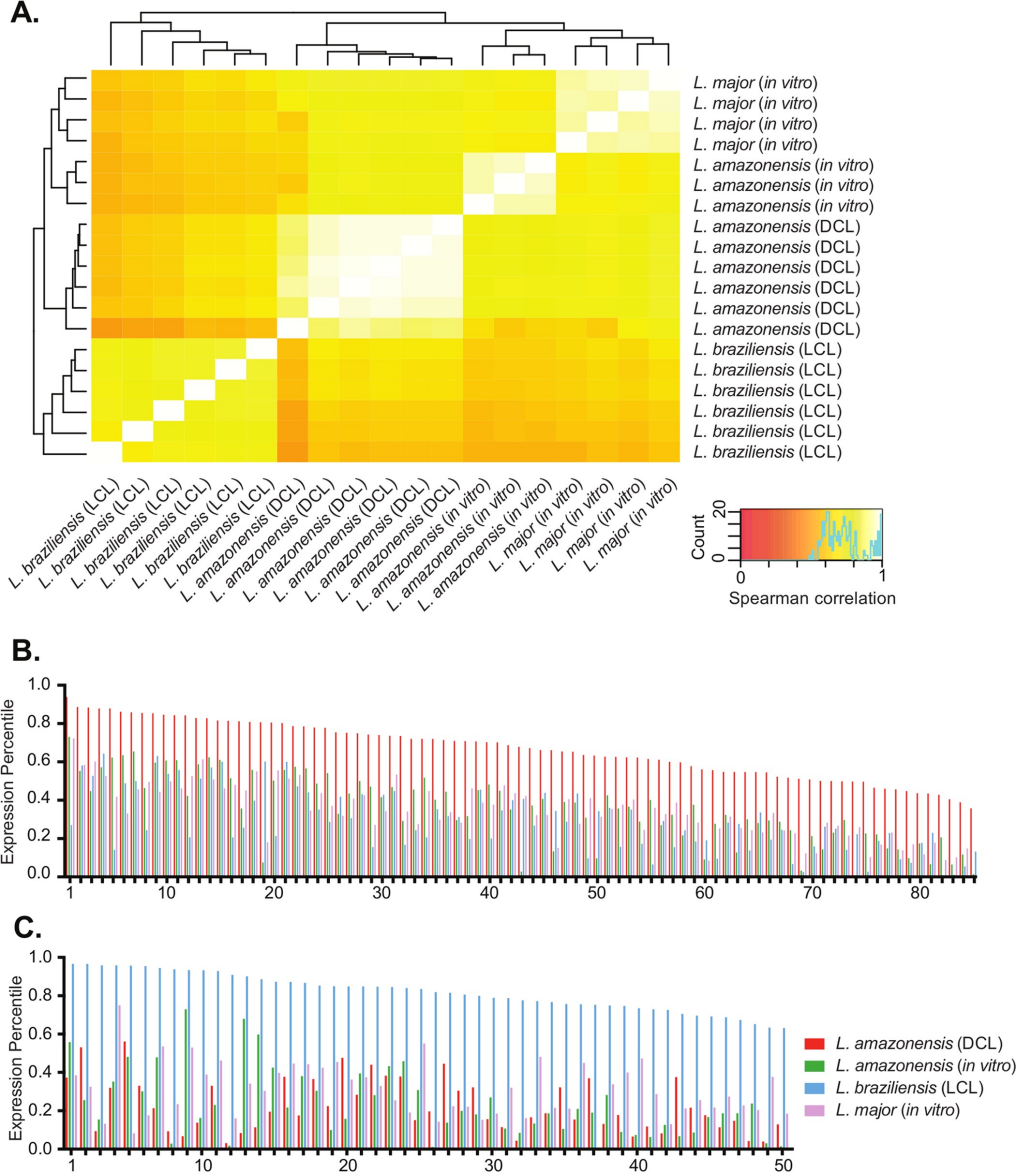
Comparisons of parasite transcriptomes in leishmaniasis and *in vitro* infections. Comparisons were made using 7272 orthologous groups present in *L*. *amazonensis* in DCL, *L*. *amazonensis* during *in vitro* macrophage infection (72hrs), *L*. *major* during *in vitro* macrophage infection (72hrs), and *L*. *braziliensis* in LCL. Orthologous group rankings were assigned using the highest expressed member of the group. **(A)** A heatmap represents the Spearman correlation of normalized orthologous group rank (by RPKM) between samples. Correlations range from 0.54 to 0.98. **(B-C)** Bars indicate orthologous group rank on a scale of 0–1 in *L*. *amazonensis* DCL (red), *L*. *amazonensis in vitro* (green), *L*. *major in vitro* (purple), and *L*. *braziliensis* LCL (blue). Expression higher in DCL (B, 85) or LCL (C, top 50 of 336) exceeded a difference greater than 0.2.

We compared parasite gene expression in DCL to gene expression in three different infection models: *in vivo L*. *braziliensis* infections in LCL [27], and *in vitro L*. *major* and *L*. *amazonensis* infections in human-cultivated macrophages [45]. In order to accurately compare gene expression across species, we used a total of 7272 orthologous groups obtained from TriTrypDB and present in all three *Leishmania* species. This included single-reciprocal orthologs as well as multi-gene families such as ribosomal and histone genes. We determined single reciprocal orthologs if only one gene was present from each species in the ortholog group. For each multi-gene ortholog group, we extracted the highest expressed gene within the group in each species to use as an indicator of expression level. Spearman correlations of all 7272 orthologous groups revealed a high degree of uniformity within each experimental condition (Fig 5A).

The deficiency in T_H_1 immune responses in DCL and the high parasite transcript expression in diffuse lesions suggested that intramacrophage parasite growth in DCL may be similarly permissive to *in vitro* parasite growth in tissue-cultured macrophages in the absence of T cells. We conversely reasoned that the strong T_H_1 response in LCL would exert immune pressure on *L*. *braziliensis* parasites resulting in an alteration in parasite transcriptional responses. Spearman correlations agreed with our hypotheses and demonstrated a high degree of similarity between DCL parasite gene expression and that of *in vitro* infections in macrophages regardless of species (Fig 5A). The *L*. *braziliensis* parasite transcriptomes, in contrast did not correlate to the same level when compared with the rest of the models (Fig 5A).

In DCL, 85 parasite genes were expressed at a higher level (expression percentile difference > 0.2) relative to parasites in the other three experimental models (Fig 5B). Of the 85 genes uniquely upregulated in DCL, 38 encoded hypothetical, proteins with no known conserved domains (S6 Table) and 3 were potential parasite virulence factors: a cyclophilin [46], a protein with leucine rich repeats [47], and a protein with a PKC phosphorylation site [48]. We also identified 195 parasite genes expressed at a significantly lower levels (difference > 0.2) in DCL, 16 of which were hypothetical proteins with no known conserved domains (S6 Table). At least 4 potential or known virulence factors were uncovered in this gene set, including a putative ecotin protein [49], ama1 protein [50], cysteine peptidase [51], and a GDP-mannose pyrophosphorylase [52].

We examined parasite gene expression unique to LCL manifestations of disease and identified 336 orthologous groups expressed at a higher level in LCL (percentile difference > 0.2), 50 of which are shown in Fig 5C (S7 Table). Of the 336, 110 were hypothetical, conserved proteins containing no known conserved domains. We found 7 potential virulence factors and 4 stress response genes, including GP63 [53], proteins containing leucine-rich repeats [47], a cyclophilin protein [46], a protein containing a RmlC-like jelly roll fold domain [54], and a Betv1-like superfamily protein [55].

**Fig 6:**
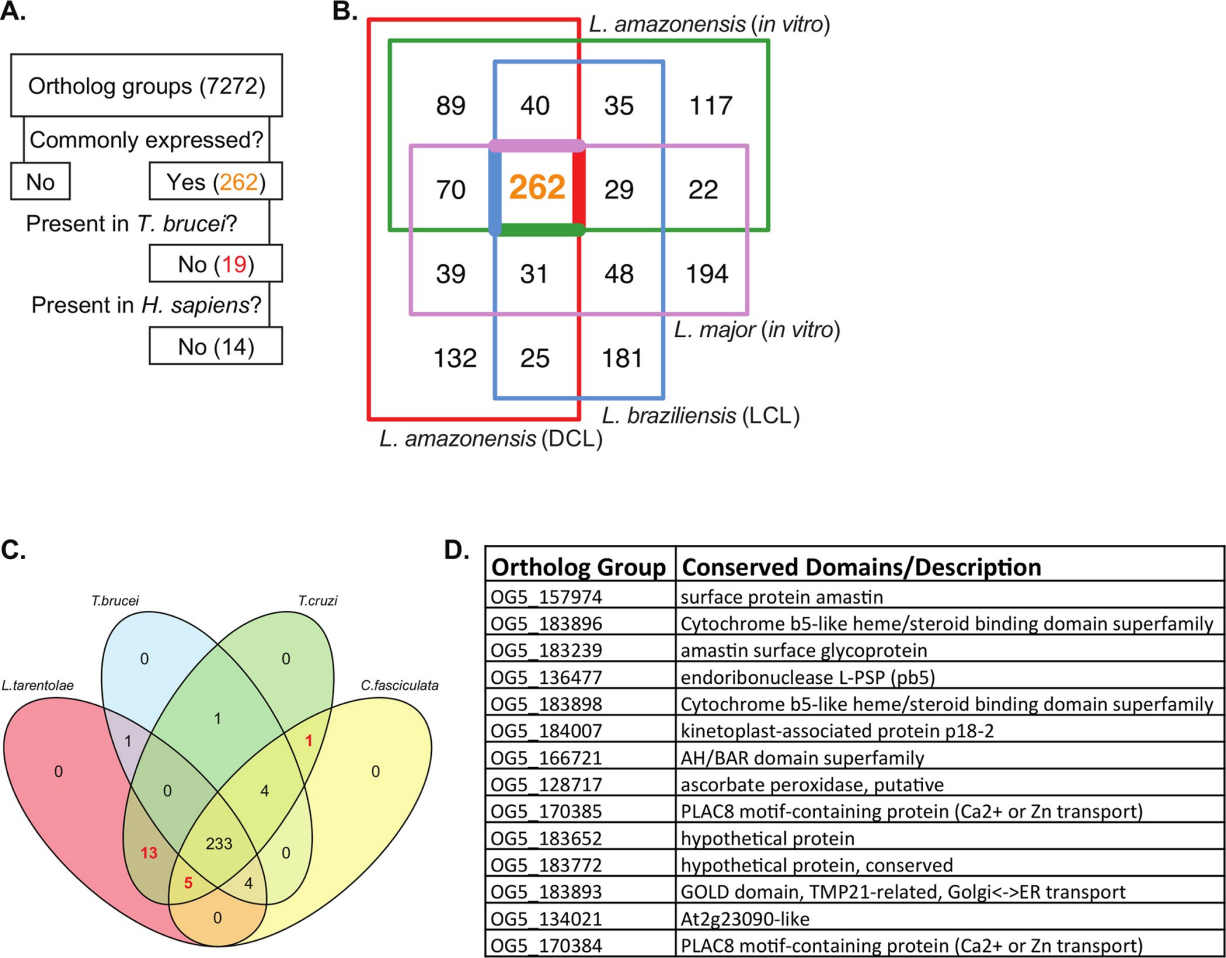
Common expression of parasite transcriptomes in leishmaniasis and *in vitro* infections. (**A)** A flowchart depicts the steps taken to identify groups of interest. **(B)** A Venn diagram of the top 10% of orthologous groups expressed (by average RPKM) in *L*. *amazonensis* in DCL (red box, 688), *L*. *amazonensis in vitro* (green box, 664), *L*. *major in vitro* (purple box, 695), and *L*. *braziliensis* in LCL (blue box, 651). The highlighted 262 orthologous groups were commonly expressed within the top 10%. **(C)** A Venn diagram explores the presence of the 262 ortholog groups (panel B) in four related trypanosomatids: *T*. *brucei*, *T*. *cruzi*, *C*. *fasciculata*, and *L*. *tarentolae*. **(D)** Table of the 14 ortholog groups extracted using the process described in panel A.

We undertook a three step process to identify a small subset of parasite genes that could prove useful in targets for therapeutic and/or vaccine research (Fig 6A). We first extracted orthologous genes commonly expressed in all four Leishmania infection models to narrow the list to highly expressed parasite genes regardless of species and condition. Next, we identified orthologous groups present in the related intracellular trypanosomatids *Trypanosoma cruzi* and *Leishmania tarentolae*, but not present in the extracellular parasite *Trypanosoma brucei*, pinpointing genes specific to intracellular parasites. Lastly, we extracted the orthologous groups not present in *Homo sapiens* to prevent overlap between parasite and host. There were 262 orthologous genes that were highly expressed by all three *Leishmania* species in the four infection models (Fig 6B, and S8 Table). Many of the genes within this subset were housekeeping genes, including 77 ribosomal proteins, 5 histone proteins, and multiple proteins related to parasite transcription or translation machinery. We also encountered 14 known or potential virulence factors, including PKC-interacting proteins [48], cysteine peptidases [51,56], cyclophilins [46], a macrophage migration inhibitory factor-like protein [57], stress response proteins [58,59], and a small myristoylated protein-3 [60] (S8 Table). Among these 262 shared genes, there were 42 that encoded hypothetical proteins with no characterized conserved domains and 46 proteins with at least one transmembrane region. Nineteen of the 262 commonly expressed orthologous groups were present in (intracellular) *T*. *cruzi* and *L*. *tarentolae* but not in (extracellular) *T*. *brucei* (Fig 6C, red numbers). Finally, 14 of those 19 were not present in *H*. *sapiens*. These 14 intracellular parasite-specific genes are listed in Fig 6D.

## Discussion

**Fig 7:**
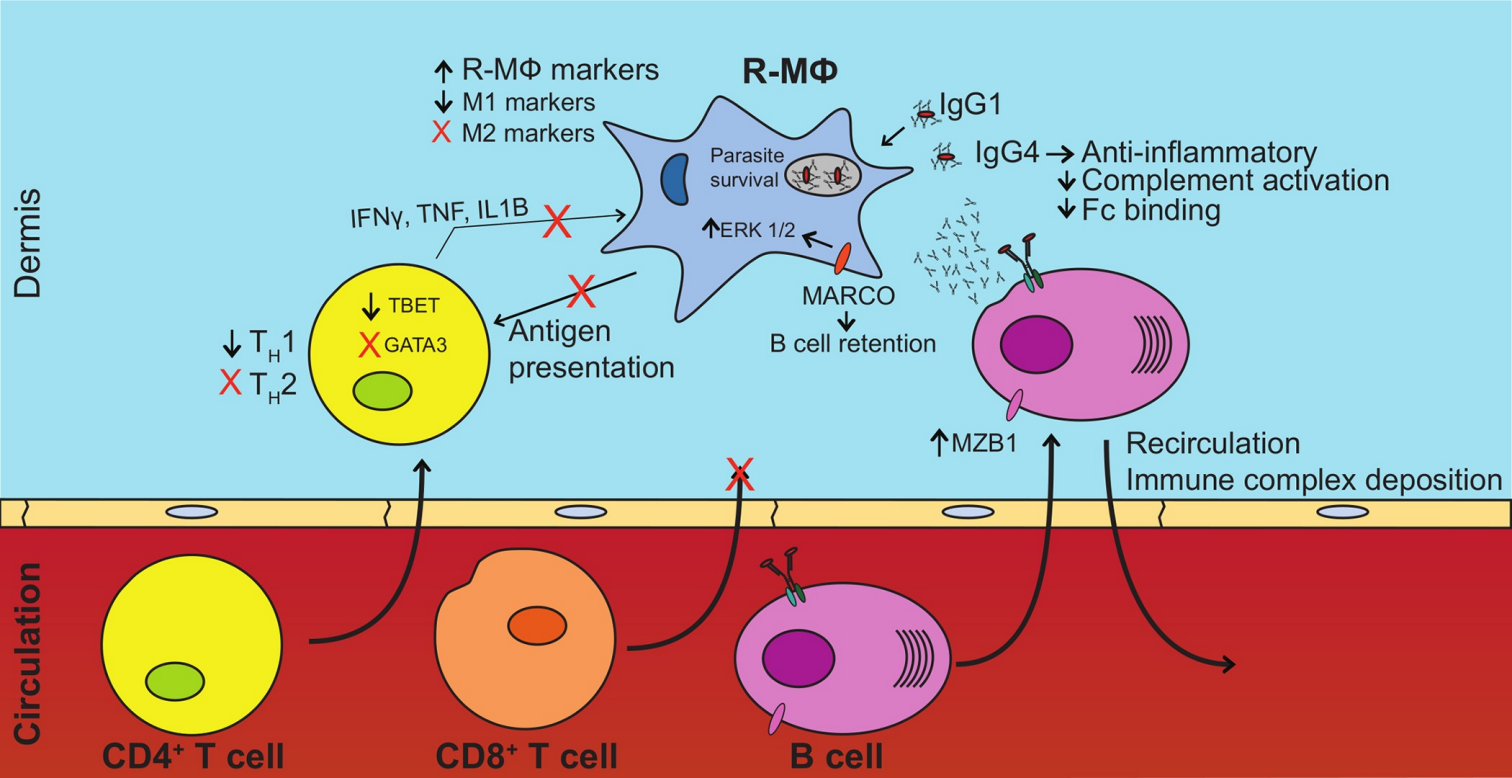
Biased B cell responses and altered macrophage and T cell activation lead to DCL phenotypes. Aspects of B cell, T cell, and macrophage responses contribute to parasite survival in DCL patients. Increased B cell presence as well as domination of the immunoglobulin repertoire by IgG4 promotes anti-inflammatory phenotypes in infected macrophages and the surrounding microenvironment, promoting regulatory macrophage phenotypes. This nullifies parasite killing, augments extracellular matrix remodeling and angiogenesis, and decreases antigen presentation. Regulatory macrophages expressing MARCO receptor supplement the anti-inflammatory macrophage response via ERK1/2 signaling and retain plasma B cells. Increased anti-inflammatory factors negate T_H_1 cell activation, cytotoxic T cell infiltration, activation, and T cell effector functions necessary for clearance of intracellular pathogens.

This work presents an in-depth assessment of the host and parasite transcriptomes in the rare disease diffuse cutaneous leishmaniasis. We identified a combination of unexpected host responses associated with disease progression in DCL. Our observations highlight a prominent role for B cells and their products in progressive disease. They also point to a diminished cytotoxic T cell response and a disease-promoting macrophage activation state. We propose that in DCL lesions elevated B cells and localized antibody production help to initiate a regulatory macrophage phenotype that is permissive to parasite growth (Fig 7).

The role of B cells in leishmaniasis appears to be complex, with published examples of their contributions to host protection or conversely to parasite persistence [11,12,19,61–63]. Our previous work in the murine system demonstrated that parasite-specific immunoglobulins failed to protect mice, and actually promoted parasite persistence in B cell deficient mice [12]. We recently demonstrated that increased immunoglobulin levels correlated with increased parasite transcripts in American tegumentary leishmaniasis [27]. Here we characterize the B cell response in human DCL and demonstrate that high levels of immunoglobulins are predictive of severe disease and could strongly contribute to it. In contrast to most other chronic infections [60], DCL lesions are dominated by IgG4 (Fig 1). IgG4 is the rarest IgG isotype, typically comprising only 1% of total IgG in normal adults [64]. In asthma, IgG4 levels are elevated and correlated with eosinophilia and IgE [65], two characteristics that were not observed in DCL lesions. The IgG4 isotype may fail to contribute to host protection because it does not bind complement or to activating FcγRs well [66–68], but rather binds to the inhibitory FcγRIIB better than the other IgG isotypes [66]. IgG4 has previously been implicated in other disease states resulting in fibroinflammatory conditions [69]. Therefore IgG4 may contribute to the anti-inflammatory and DTH-refractory nature of the disease [70–72]. This anomaly was observed 23 years ago in the serum of DCL patients [73]. Here, we demonstrate that the rare IgG4 isotype permeates lesions as well.

Another unexpected feature of B lymphocytes in DCL is that they share markers with marginal zone B cells. This could explain their retention in lesions and the diffuse nature of the disease. Upregulation of MZB1 in DCL (500-fold) was unexpected in skin and we observed other transcriptional signatures common to MZ B cells in DCL, including an upregulation of complement receptors (CD1D, CD21, and CD35; S2 Table) and CD27 (Fig 1D) and a lack of germinal center markers (BCL6, CD10 not differentially expressed) [74–79]. MZ B cells have a lower activation threshold than follicular B cells, to permit the rapid initiation of IgM production and IgG- and IgA class-switch recombination (CSR) in the absence of CD40-dependent help from T follicular helper (TFH) cells. Moreover, MZ B cells can respond to blood-borne thymus-independent (TI) antigens, help initiate APC uptake of antigen [78], produce cytokines, and contribute to tissue repair [74,80,81]. Schiller et al. discovered these cells in fibrotic skin and lung tissue, exposing their ability to contribute to fibrotic disease [82]. The recirculating tendencies of human “memory”-like MZ B cells [75] could explain the phenotype of uncontrolled lesion development in later stages of DCL.

Immunoglobulin sequencing analysis indicated skewed usage of specific V genes in heavy and light chain immunoglobulins (S2 and S3 Figs). Analysis of V-J combinations in DCL patients revealed a relatively restricted subset of gene usage, suggestive of an oligoclonal, antigen-driven response (S3 Fig). However, we do not know if these are T-independent, innate-like or T-dependent, class-switched B cells. Further analysis will be needed to fully understand the immunoglobulin responses and variable gene selection processes in diffuse lesions.

We also identified a change in the T cell population that could contribute to parasite survival and disease progression. It is widely accepted that in murine models of leishmaniasis, a T_H_1 response is responsible for protection against *Leishmania major*, while a T_H_2 response leads to parasite persistence [83,84]. In DCL lesions, we observed approximately a 6-fold increase in transcripts for CD4 compared to healthy skin, a magnitude similar to that previously observed in LCL [27]. However, analysis of T_H_1 effectors and the transcription factor T-bet (Fig 2) demonstrated a diminished T_H_1 response in DCL relative to LCL. Importantly, at the time of this analysis, years after the initiation of disease, the decreased T_H_1 transcriptional response in DCL was not compensated for by an increase in T_H_2-associated transcripts. Neither LCL nor DCL lesions exhibited signs of a T_H_2 transcriptional response (Fig 2B, S4 Fig), which was unexpected given previous work in patients with DCL [85,86]. Transcripts for the canonical T_H_2 cytokines, IL-4, IL-5, and IL-13, and the master T_H_2 transcription factor, GATA-3, were virtually absent in DCL at the time the biopsies were taken, indicating that the sustained production of T_H_2 cytokine transcripts may not be required for the maintenance of this disease.

The role of cytotoxic CD8^+^ T cells in leishmaniasis appears to be complex [2]. Cytotoxic responses in LCL can contribute to the control of parasite growth but they may also contribute to tissue destruction and ulceration of the lesion [2]. We hypothesize that the paucity of CTL cytotoxicity in DCL may prevent ulceration but allow for prolonged survival of infected macrophages, thereby promoting parasite survival [87,88]. This increased survival would be consistent with the high percentage of transcripts mapping to the parasite genome observed in Fig 4. The relative lack of CD8 transcripts in DCL, combined with a marked decrease in transcripts for perforin, granzymes, and granulysin (Fig 2D) is consistent with a decreased cytotoxic T cell response in DCL.

In our previous work on LCL, we demonstrated host responses indicative of classically-activated macrophages, with substantial upregulation of IDO1, CXCL9, CXCL10, CXCL11, GBP5, IL-6, and CCL8 [27]. In the present work, we find that classical macrophage activation markers were significantly decreased in DCL lesions (Fig 3). We hypothesized that high IgG in lesions would promote the development of a regulatory macrophage population in lesions. Indeed, an upregulation of anti-inflammatory and angiogenic gene transcription in DCL lesions (Fig 3) is consistent with this hypothesis. Expression of MARCO on macrophages could also contribute to parasite persistence through retention of MZB1^+^ B cells [81,89–91] and enhanced ERK1/2 signaling known to exacerbate disease [92,93]. The presence of regulatory macrophages in DCL would contribute to parasite persistence as intracellular pathogen killing is diminished in this macrophage subset [94].

An analysis of the parasite transcriptome in lesions allowed for the identification of parasite genes that could potentially contribute to disease pathology. The uniformity of parasite transcription among the six patients was quite remarkable (Fig 4) despite differences in patient age (15–50) and length of infection (14–35 years). More importantly, we combined our most recent data from DCL parasites with previous parasite transcriptome datasets to narrow the list of targets for disease causation. Previous research suggested that differences in disease manifestation are due primarily to changes in the host [95]. More recent studies have implicated specific parasite gene contributions to pathogenesis [56,96,97]. We identified some 280 parasite orthologous groups expressed at a different level in DCL infections compared to other model infections (S6 Table, Fig 5B). Virulence factors expressed at a higher level in DCL, such as cyclophilins [46], PKC-interacting proteins [48], or leucine-rich repeat containing proteins [47] could act as mitogens that influence immune responses. Antigens unique to DCL could contribute to the disease phenotype by inducing the specific IgG4 responses we observe in DCL patients. Conversely, genes expressed at a lower level in DCL (S6 Table) could contribute to the paucity in CD8^+^ T cell recruitment and lower CD4^+^ T_H_1 cell activation. For example, diminished presence of potential or known virulence factors like cysteine peptidase [51], ama1 protein [50], GDP-mannose pyrophosphorylase [52], or ecotin [49] in DCL could significantly decrease immunogenicity or T cell activation within the lesion microenvironment.

A comparison of parasite gene expression in LCL lesions with several other leishmania infection models (Fig 5A) revealed 336 parasite genes expressed at higher levels in LCL (S7 Table). These *L*. *braziliensis* genes could either be contributing to the hyperinflammatory nature of LCL, or they could be a result of increased immune pressure in the lesion microenvironment. Of note are the known virulence factors GP63 [53], a cyclophilin [46], and multiple leucine-rich repeat domain containing proteins [47]. These lists should be a starting point for understanding host-pathogen interactions, parasite manipulation of host responses, and parasites responses to immune pressure.

Lastly, we identified a panel of 262 parasite ortholog groups that are highly expressed regardless of disease manifestation, condition and species (Fig 6). Many of the proteins encoded for by these genes have unknown structures and functions (S8 Table), indicating the need for continued research and annotation of parasite genomes. Research on the roles of the 14 virulence factors in this group is already underway and here we highlight the need to expand our knowledge. The 14 genes that are not present in the *T*. *brucei* or human genome (Fig 6D) could shed light on disease pathogenesis and parasite intracellular survival.

In conclusion, we used high-throughput sequencing to simultaneously characterize host immune responses and parasite gene expression in human diffuse cutaneous leishmaniasis. These lesions lack a DTH^+^ response and are generally pain-free, but are disfiguring and spread over most of a patient’s body. Our analysis of host transcriptomes demonstrated an expected reduction of inflammatory responses and signaled the existence of regulatory macrophages that are unable to kill parasites. We believe the DCL pathology is a result of improperly biased B cell responses that lead to dampened macrophage inflammation, coupled with a lack of CD8^+^ T cell cytotoxicity. The infiltration of atypical B cells and increased IgG4 production demonstrate a possible role in shifting the immune response away from T_H_1 environments necessary for parasite killing and infection resolution. In macrophages, we observe augmented immunoregulatory and anti-inflammatory responses coupled with angiogenesis, reorganization of extracellular matrix, and flourishing parasite growth. Parasite manipulation of the host immune response may also occur, and have identified parasite genes that may contribute to the diffuse nature of this disease. We also identified conserved parasite gene expression across multiple species and conditions. These studies on parasite gene expression may reveal new targets for vaccine development in this neglected tropical disease.

## Supporting information

S1 Fig The human host transcriptome in L. amazonensis-infected DCL patients (A) A principal component analysis plot demonstrates wholetranscriptome differences between healthy (grey), LCL (blue), and DCL (red). Principal component 1 represents 44.43% of the variance and principal component 2 represents 14.95%. (B) A heatmap shows the correlation between human host gene expression among the 6 DCL patients (C-D) Venn diagrams show up (E) and downregulated (F) genes in DCL (red) and LCL (blue) circles compared to healthy skin.
(PDF)

S2 Fig Immunoglobulin repertoires in DCL patients are oligoclonal. A representative chord diagram shows average V and J gene usage (width of gene arc) and combination frequency (width of ribbons) in DCL patients.
(PDF)

S3 Fig Skewed immunoglobulin V gene and subgroup usage in DCL lesions. Heavy and light chain V gene subgroup and gene usage frequency shows skewed usage of V genes, limited to 25 heavy (Panel A), 22 kappa (Panel B), and 18 lambda (Panel C) genes with a frequency greater than 1%.
(PDF)

S4 Fig Minimal TH2/M2a responses in LCL and DCL lesions. (A) Bars show log2 fold-changes of various TH2 and M2a markers and effector molecules. Of 26, 14 were upregulated in LCL (blue), 13 were upregulated in DCL (red), 2 were downregulated in LCL, and 2 were downregulated in DCL. Only 5 demonstrated significant differences (*, p < 0.05) between LCL and DCL (CCR4, IRF4, FGL2, CCL14, CCL26). (B) Bars show RPKMs for each of the TH2/M2a-related genes. Only 3 genes exceeded RPKMs of 30.
(PDF)

S1 Table Experimental design. Table of sample IDs, mapping statistics, and patient data.
(XLSX)

S2 Table Top upregulated genes in DCL vs. healthy controls. Log2 fold-changes of the top 100 upregulated genes in DCL compared to healthy plus three additional MZ B cell genes.
(XLSX)

S3 Table M1 Markers downregulated in DCL vs. LCL. Log2 fold-changes of M1 markers in LCL and DCL compared to healthy and each other.
(XLSX)

S4 Table Regulatory macrophage markers upregulated in DCL vs LCL. Log2 fold-changes of regulatory macrophage markers in LCL and DCL compared to healthy and each other.
(XLSX)

S5 Table Top parasite genes expressed in DCL. Rank, mean RPKM, and standard error of the mean for the top parasite genes expressed in DCL.
(XLSX)

S6 Table Genes unique to DCL (DCL higher, DCL lower). Description and ranking of parasite genes expressed at a higher or lower level in DCL compared to LCL or *in vitro* experiments.
(XLSX)

S7 Table Genes unique to LCL (LCL higher). Description and ranking of parasite genes expressed at a higher level in LCL compared to DCL or *in vitro* experiments.
(XLSX)

S8 Table Pan-*Leishmania* markers. Description and ranking of parasite genes expressed at a high level in all experiments.
(XLSX)
