# Supplementary figures and images for: Host and parasite responses in human diffuse cutaneous leishmaniasis caused by L. amazonensis
Source: PLoS Negl Trop Dis. 2019 Mar 7;13(3):e0007152. doi: 10.1371/journal.pntd.0007152 (PMC6405045; doi:10.1371/journal.pntd.0007152)

**A.**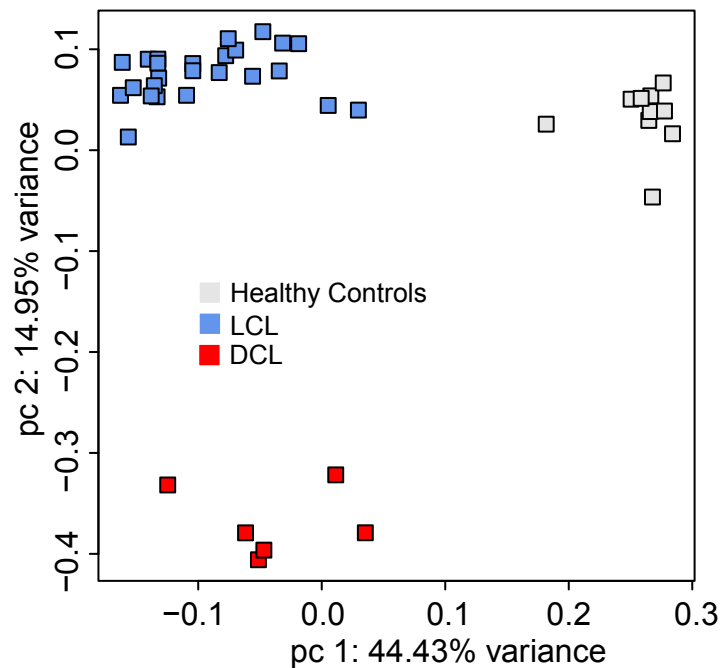**B.**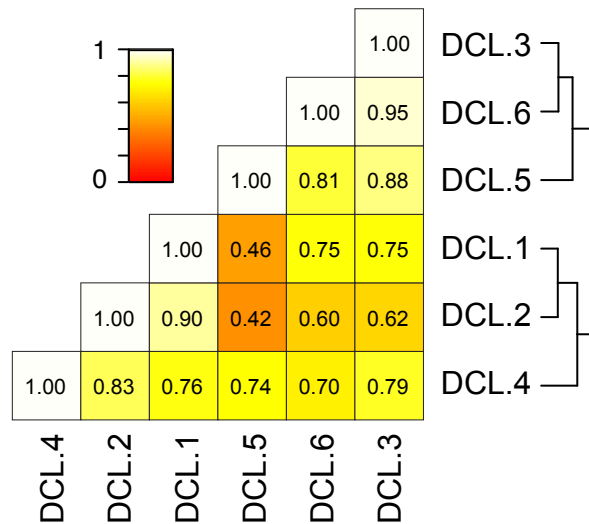**C.**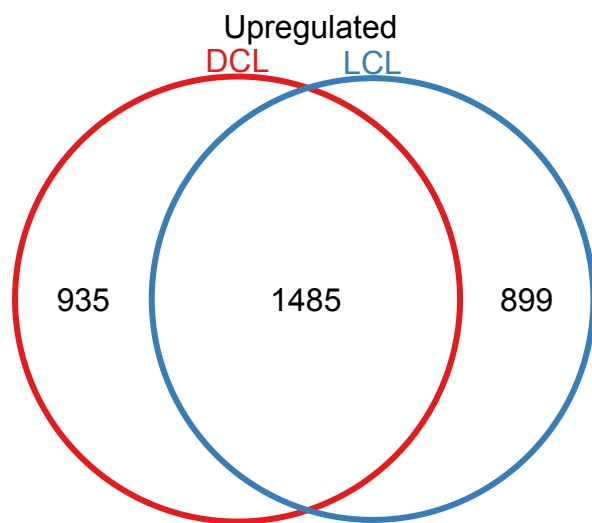**D.**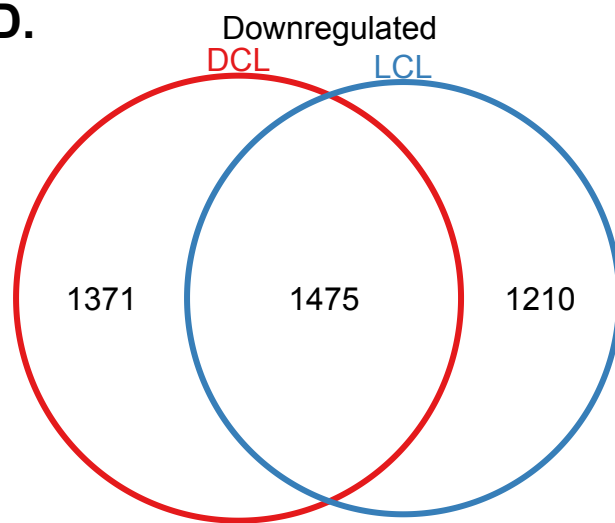

Supplement: S1 Fig — The human host transcriptome in L. amazonensis-infected DCL patients (A) A principal component analysis plot demonstrates wholetranscriptome differences between healthy (grey), LCL (blue), and DCL (red). Principal component 1 represents 44.43% of the variance and principal component 2 represents 14.95%. (B) A heatmap shows the correlation between human host gene expression among the 6 DCL patients (C-D) Venn diagrams show up (E) and downregulated (F) genes in DCL (red) and LCL (blue) circles compared to healthy skin. (PDF) [file pntd.0007152.s001.pdf]

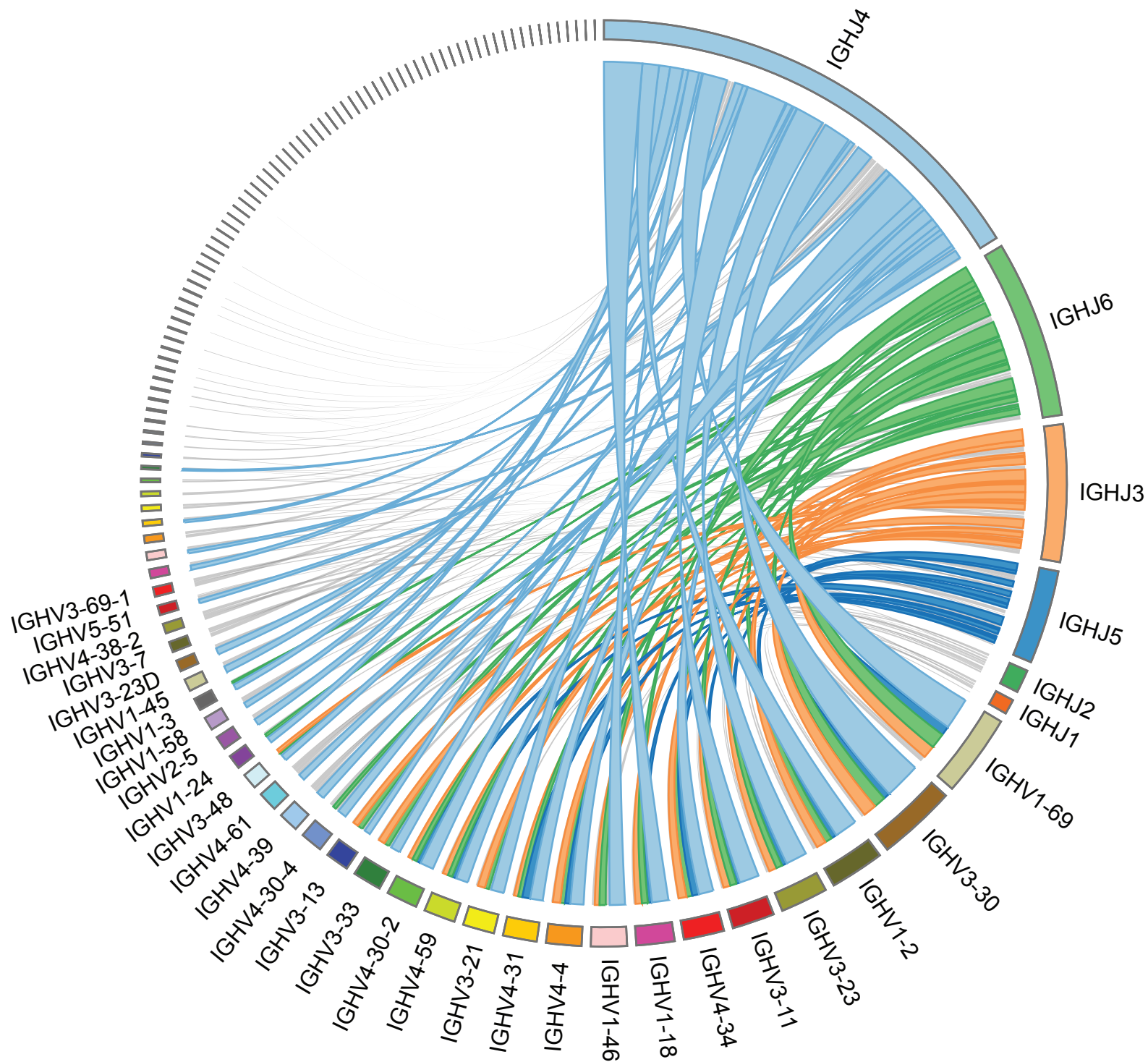

Supplement: S2 Fig — A representative chord diagram shows average V and J gene usage (width of gene arc) and combination frequency (width of ribbons) in DCL patients. (PDF) [file pntd.0007152.s002.pdf]

**A.**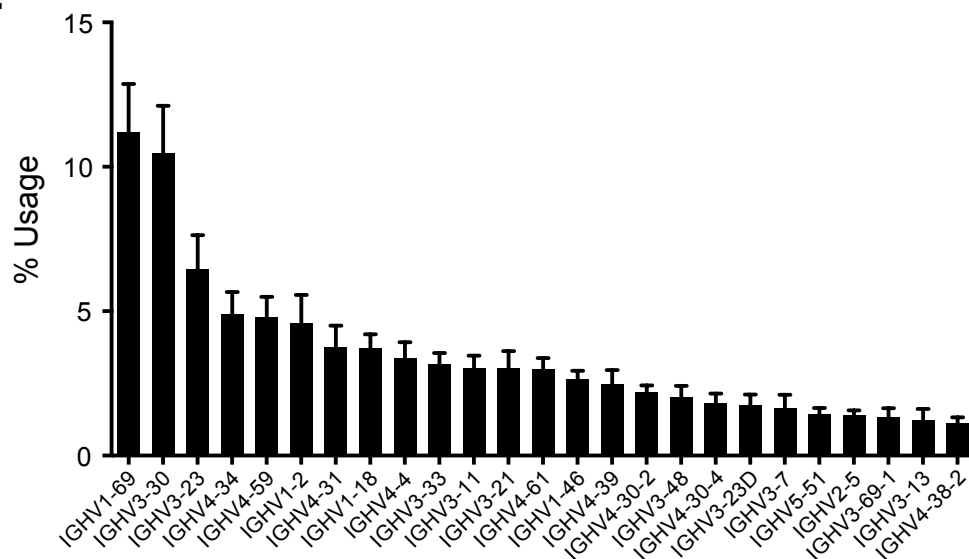**B.**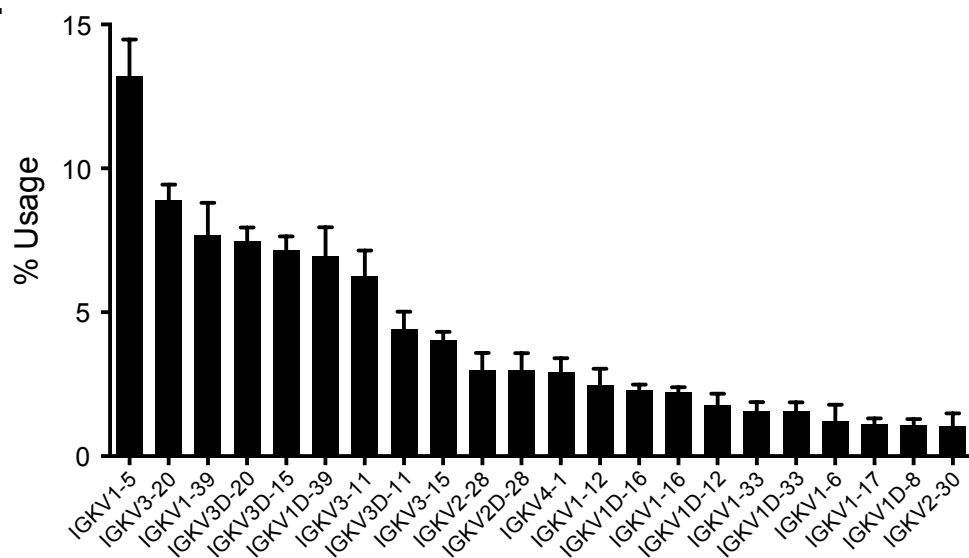**C.**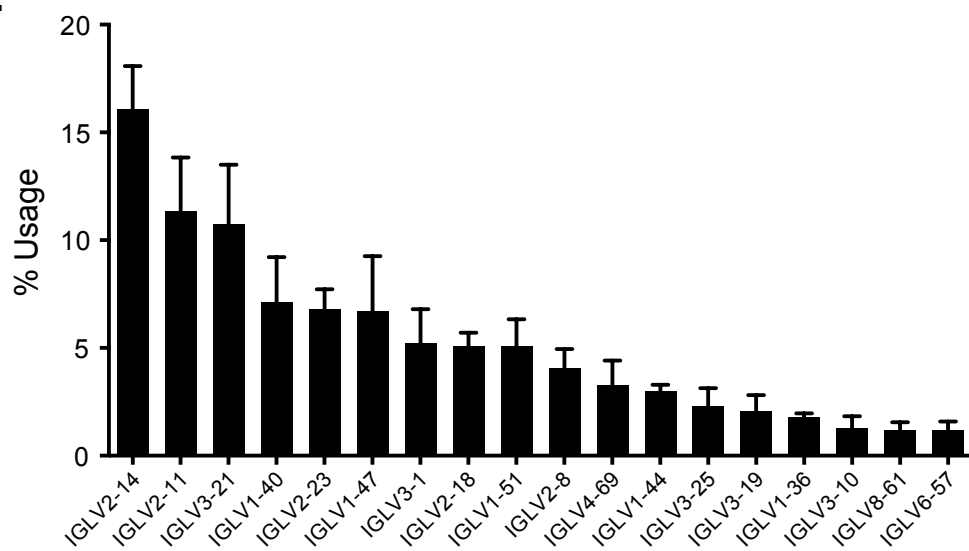

Supplement: S3 Fig — Heavy and light chain V gene subgroup and gene usage frequency shows skewed usage of V genes, limited to 25 heavy (Panel A), 22 kappa (Panel B), and 18 lambda (Panel C) genes with a frequency greater than 1%. (PDF) [file pntd.0007152.s003.pdf]

**A.**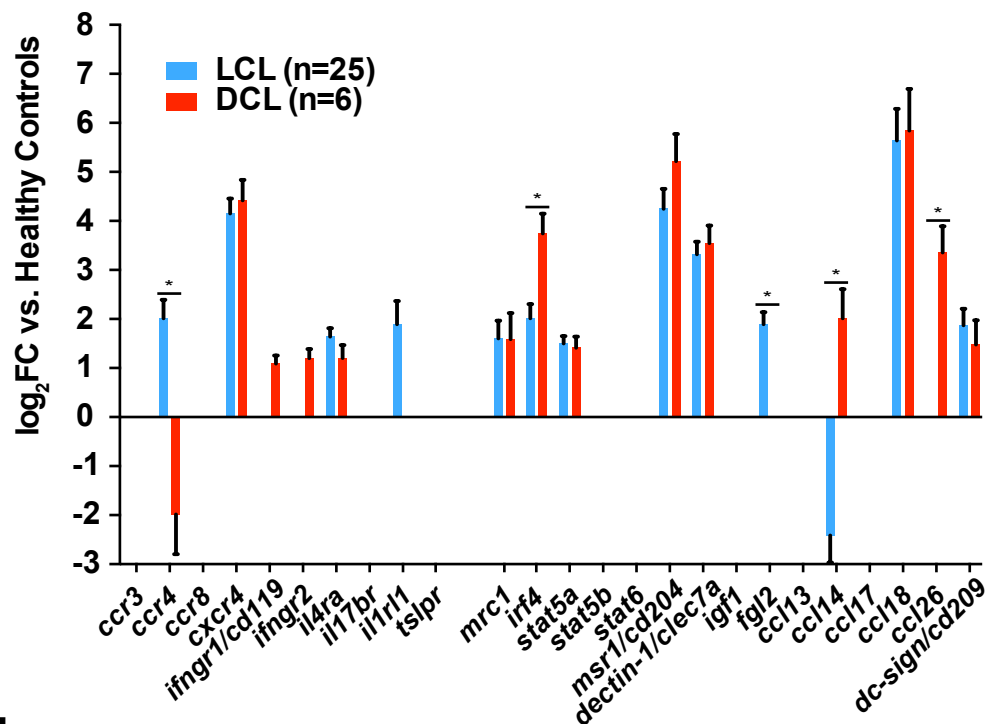**B.**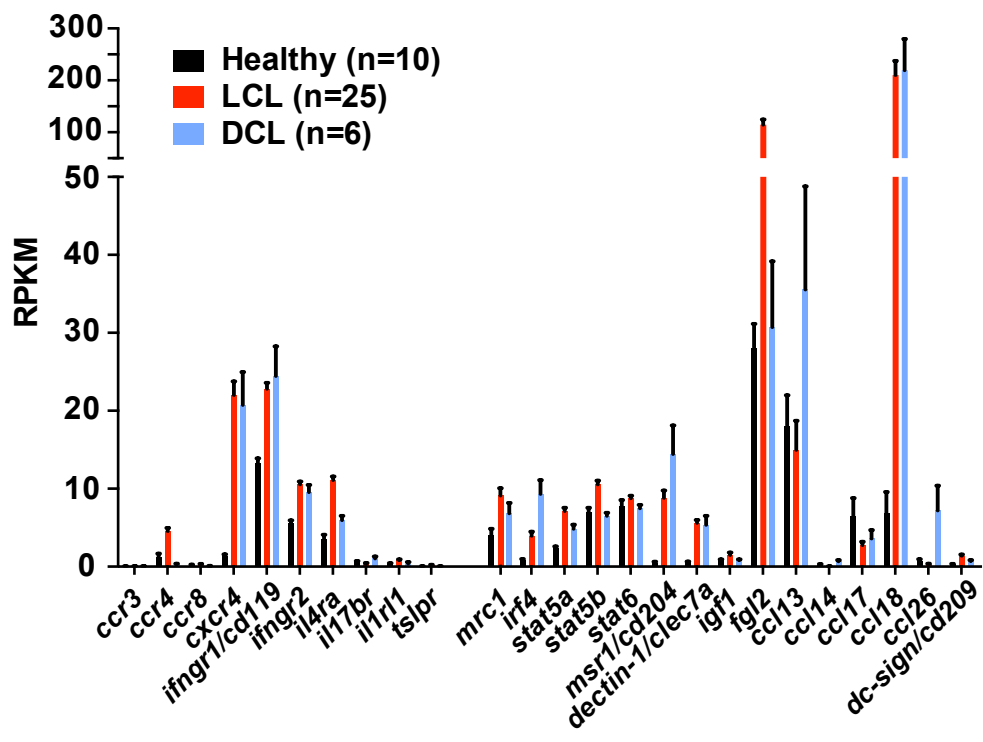

Supplement: S4 Fig — (A) Bars show log2 fold-changes of various TH2 and M2a markers and effector molecules. Of 26, 14 were upregulated in LCL (blue), 13 were upregulated in DCL (red), 2 were downregulated in LCL, and 2 were downregulated in DCL. Only 5 demonstrated significant differences (*, p < 0.05) between LCL and DCL (CCR4, IRF4, FGL2, CCL14, CCL26). (B) Bars show RPKMs for each of the TH2/M2a-related genes. Only 3 genes exceeded RPKMs of 30. (PDF) [file pntd.0007152.s004.pdf]
